# The Presence of Serum TgAb Suggests Lower Risks for Glucose and Lipid Metabolic Disorders in Euthyroid General Population From a National Survey

**DOI:** 10.3389/fendo.2020.00139

**Published:** 2020-03-18

**Authors:** Jinjia Zhang, Yiyang Gao, Yongze Li, Di Teng, Yuanming Xue, Li Yan, Jing Yang, Lihui Yang, Yongli Yao, Jianming Ba, Bing Chen, Jianling Du, Lanjie He, Xiaoyang Lai, Xiaochun Teng, Xiaoguang Shi, Yanbo Li, Haiyi Chi, Eryuan Liao, Chao Liu, Libin Liu, Guijun Qin, Yingfen Qin, Huibiao Quan, Bingyin Shi, Hui Sun, Xulei Tang, Nanwei Tong, Guixia Wang, Jin-an Zhang, Youmin Wang, Zhen Ye, Qiao Zhang, Lihui Zhang, Jun Zhu, Mei Zhu, Weiping Teng, Zhongyan Shan, Jing Li

**Affiliations:** ^1^Department of Endocrinology and Metabolism, Institute of Endocrinology, The First Affiliated Hospital of China Medical University, Shenyang, China; ^2^Department of Endocrinology, The First People's Hospital of Yunnan Province, Kunming, China; ^3^Department of Endocrinology and Metabolism, Sun Yat-sen Memorial Hospital, Sun Yat-sen University, Guangzhou, China; ^4^Department of Endocrinology, The First Hospital of Shanxi Medical University, Taiyuan, China; ^5^Department of Endocrinology and Metabolism, People's Hospital of Tibet Autonomous Region, Lhasa, China; ^6^Department of Endocrinology, Qinghai Provincial People's Hospital, Xining, China; ^7^Department of Endocrinology, Chinese PLA General Hospital, Beijing, China; ^8^Department of Endocrinology, Southwest Hospital, Third Military Medical University, Chongqing, China; ^9^Department of Endocrinology, The First Affiliated Hospital of Dalian Medical University, Dalian, China; ^10^Department of Endocrinology, Cardiovascular and Cerebrovascular Disease Hospital of Ningxia Medical University, Yinchuan, China; ^11^Department of Endocrinology and Metabolism, The Second Affiliated Hospital of Nanchang University, Nanchang, China; ^12^Department of Endocrinology, The First Affiliated Hospital of Harbin Medical University, Harbin, China; ^13^Department of Endocrinology, Hohhot First Hospital, Hohhot, China; ^14^Department of Endocrinology and Metabolism, The Second Xiangya Hospital, Central South University, Changsha, China; ^15^Research Center of Endocrine and Metabolic Diseases, Affiliated Hospital of Integrated Traditional Chinese and Western Medicine, Nanjing University of Chinese Medicine, Nanjing, China; ^16^Department of Endocrinology and Metabolism, Fujian Institute of Endocrinology, Fujian Medical University Union Hospital, Fuzhou, China; ^17^Division of Endocrinology, Department of Internal Medicine, The First Affiliated Hospital, Zhengzhou University, Zhengzhou, China; ^18^Department of Endocrine, First Affiliated Hospital of Guangxi Medical University, Nanning, China; ^19^Department of Endocrinology, Hainan General Hospital, Haikou, China; ^20^Department of Endocrinology, The First Affiliated Hospital of Xi'an Jiaotong University, Xi'an, China; ^21^Department of Endocrinology, Union Hospital, Tongji Medical College, Huazhong University of Science and Technology, Wuhan, China; ^22^Department of Endocrinology, The First Hospital of Lanzhou University, Lanzhou, China; ^23^State Key Laboratory of Biotherapy, Department of Endocrinology and Metabolism, West China Hospital, Sichuan University, Chengdu, China; ^24^Department of Endocrinology and Metabolism, The First Hospital of Jilin University, Changchun, China; ^25^Department of Endocrinology, Shanghai University of Medicine & Health Science Affiliated Zhoupu Hospital, Shanghai, China; ^26^Department of Endocrinology, The First Hospital of Anhui Medical University, Hefei, China; ^27^Zhejiang Provincial Center for Disease Control and Prevention, Hangzhou, China; ^28^Department of Endocrinology and Metabolism, Affiliated Hospital of Guiyang Medical University, Guiyang, China; ^29^Department of Endocrinology, Second Hospital of Hebei Medical University, Shijiazhuang, China; ^30^Department of Endocrinology, The First Affiliated Hospital of Xinjiang Medical University, Ürümqi, China; ^31^Department of Endocrinology and Metabolism, Tianjin Medical University General Hospital, Tianjin, China

**Keywords:** glucose, lipid, TPOAb, TgAb, IFG, hypertriglyceridemia

## Abstract

**Purpose:** The expressions of antibodies against thyroid peroxidase (TPOAb) and thyroglobulin (TgAb) are very common in the sera of patients with autoimmune thyroid diseases (AITD). The relationship between thyroid autoantibodies and the occurrence of glucose and lipid metabolic disorders remains unclear. This study was performed to investigate the correlation between the presence of serum TPOAb/TgAb and those metabolic disorders in euthyroid general population.

**Methods:** The data of this study were derived from the Thyroid Disease, Iodine status, and Diabetes National epidemiological (TIDE) survey from all 31 provinces of mainland China. A total of 17,964 euthyroid subjects including 5,802 males (4,000 with TPOAb^−^TgAb^−^ and 1,802 with TPOAb^+^/TgAb^+^) and 12,162 females (8,000 with TPOAb^−^TgAb^−^ and 4,162 with TPOAb^+^/TgAb^+^) were enrolled in this study. The blood glucose and lipid levels were compared between individuals with TPOAb^−^TgAb^−^ and those with TPOAb^+^TgAb^−^, TPOAb^−^TgAb^+^, TPOAb^+^TgAb^+^.

**Results:** Both fasting blood glucose (FBG) concentration and the proportion of individuals with impaired FBG (IFG) showed the decreased trends in TPOAb^−^TgAb^+^ males as compared with TPOAb^−^TgAb^−^ men. There were significantly lower FBG and higher HDL-C levels as well as tendencies toward decreased incidences of IGT and hypertriglyceridemia in TPOAb^−^TgAb^+^ females when compared with TPOAb^−^TgAb^−^ women. Binary logistic regression analysis further showed that serum TgAb single positivity in males was an independent protective factor for IFG with an OR of 0.691 (95% CI, 0.503–0.949). For females, serum TgAb single positivity was an independent protective factor for hypertriglyceridemia with an OR of 0.859 (95% CI, 0.748–0.987). Trend test showed that with the increase of serum TgAb level, there were significant decreases in the prevalence of IFG among the men with TSH ≤ 2.5 mIU/L and that of hypertriglyceridemia in the women, especially among non-obese females.

**Conclusion:** Serum TgAb single positivity may imply a reduced risk of IFG in euthyroid men and that of hypertriglyceridemia in euthyroid women. The mechanisms for the independent protective roles of TgAb await further investigation.

## Introduction

Autoantibodies against thyroid peroxidase (TPOAb) and thyroglobulin (TgAb) are commonly found in the sera of patients with autoimmune thyroid diseases (AITD), and may contribute to the abnormal thyroid functions (1). Our surveys in 1999 and 2011 have shown that the positive rate of TPOAb in the general population was 9.81 and 11.5%, and that of TgAb was 9.09 and 12.6%, respectively (2, 3). It has been found that these thyroid autoantibodies may not only affect the thyroid tissue, but also directly act on some other organs and cause extra-thyroidal damages, such as nephropathy (4), and encephalopathy (5). However, there is a lack of investigations about the direct effects of those autoantibodies on glucose and lipid metabolisms, and the few findings are inconsistent. Chen et al. showed that the presence of thyroid autoimmunity increased the risk of metabolic disorders (6), while Agbaht et al. found that the serum TPOAb level was not associated with the occurrence of metabolic syndrome in obese people (7). Furthermore, one study from Portugal has shown that serum TPOAb positivity may be a protective factor for metabolic syndrome and hypertriglyceridemia (8). Those previous studies did neither exclude subjects with thyroid dysfunctions nor completely discriminate the individual roles of TPOAb and TgAb. It is well-known that both hyperthyroidism and hypothyroidism can directly result in glucose and lipid disorders. Thus, the current study has been designed to analyze the respective relationships of TPOAb and TgAb with the occurrence of glucose and lipid metabolic disorders in euthyroid population.

Our group has led a cross-sectional Thyroid Disease, Iodine Status, and Diabetes National Epidemiological Survey (TIDE), which was conducted nationwide in mainland China during 2015–2017, under the support of Chinese Society of Endocrinology (CSE), and Chinese Thyroid Association (CTA). This national survey investigated the iodine nutrition status and the prevalence of thyroid disorders and diabetes, as well as the relationships between them in China. We systematically analyzed the associations between serum TPOAb and TgAb expressions and the prevalence of glucose and lipid metabolic disorders in a random sample that is representative of the general population with normal thyroid functions. The sample size was larger than all previously published reports which had focused on the above issues. It also covered more representative Chinese population from the 31 provinces of mainland China.

## Materials and Methods

### Study Participants

In the TIDE program, a multi-stage, cluster-based, and stratified random sampling method was adopted to enroll nationally representative general population aged 18 years and above, and the program covered all 31 provinces of mainland China. One city and one village were selected from each province. An urban district from each city and a rural township from each village were then randomly selected. Finally, two residential communities were randomly selected from each urban district or rural township. The inclusion criteria for the participants from the total 124 communities were as followed: (1) aged 18 years and above; (2) living in the selected community for at least 5 years; (3) not receiving iodine-containing drugs or contrast agents in the past 3 months; and (4) not pregnant (for women). The research protocol was approved by the Ethics Committee of the China Medical University. After detailed explanation of the study protocol, all participants signed the informed consent.

**Figure 1:**
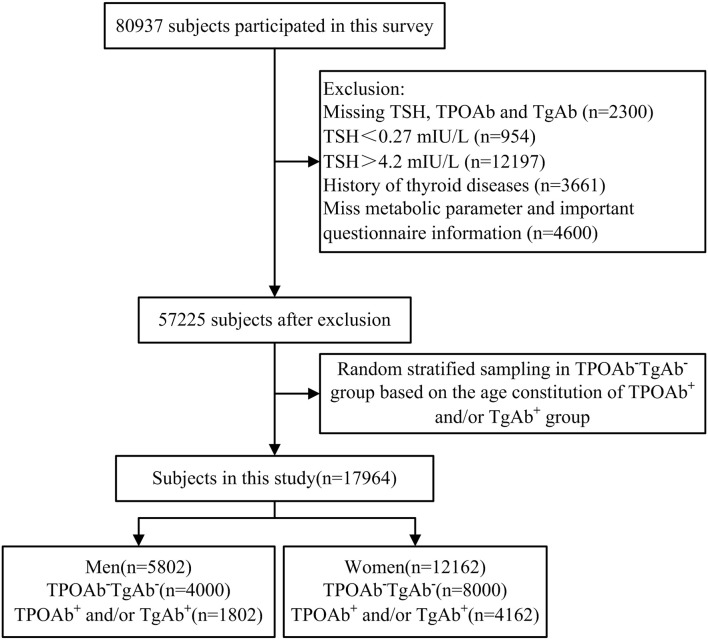
Flowchart of the inclusion and exclusion of participants in this study.

A total of 80,937 individuals participated in the TIDE program. Among them, 23,712 participants were excluded from the current study due to missing information about thyroid stimulating hormone (TSH), TPOAb, TgAb, metabolic parameters, or questionnaire information. Participants with abnormal TSH and personal history of thyroid diseases were also excluded. Finally, a total of 57,225 subjects remained. All those subjects with positive expressions of serum thyroid autoantibodies were enrolled in this current study (TPOAb^+^ and/or TgAb^+^, 1,802 males, and 4,162 females), and were assigned into three groups based on their differential expression: (1) TPOAb single positive group (TPOAb^+^TgAb^−^) (2), TgAb single positive group (TPOAb^−^TgAb^+^), and (3) TPOAb and TgAb double positive group (TPOAb^+^TgAb^+^). Based on the age-related ratio of the antibody-positive group (TPOAb^+^ and/or TgAb^+^), 1:2 random sampling after stratification by age and gender was performed from the antibody-double negative (TPOAb^−^TgAb^−^) individuals using SPSS software, and 4,000 males and 8,000 females were enrolled into the TPOAb^−^TgAb^−^ group in the current study. Finally, a total of 17,964 subjects were included in this study (Figure 1).

### Data Collection

To ensure the validity of the data and establish a high-quality control system, all investigators were trained on research programs and standard operating procedures before the start of the project, and were assessed through on-site simulation. Only those who passed the assessment participated in collecting data. Unified quality control was accepted during the investigation. All the questionnaires were administered by trained interviewers, and they were reviewed and kept by dedicated personnel on a daily basis, and errors were corrected on time. The physical examinations were performed by fixed medical staff. All the instruments were regularly inspected and calibrated. Data were inputted by two independent persons, and errors were regularly checked.

The questionnaire included demographic characteristics, personal and family medical history of thyroid diseases, smoking status, family income, education level, and household salt consumption. Location of residence included urban and rural areas, and the educational level was divided into high school and below, and college and above. Physical examination included height (accurate to 0.1 cm), body weight (accurate to 0.1 kg), waist circumference (accurate to 0.1 cm), and body mass index (BMI) calculated by weight/height^2^ (kg/m^2^). The blood pressure was determined twice after the participant had rested for 10 min at an interval of 3–5 min, and the average level was recorded. Samples of fasting venous blood and spot urine were collected from all subjects. Those who had not been diagnosed with diabetes received an oral glucose tolerance test (OGTT).

### Laboratory Tests and Thyroid Ultrasonography

After the survey was completed, all specimens collected were airlifted through a cold chain system to the institute of Endocrinology of China Medical University for centralized tests. Blood glucose (BG) was tested by the Hexokinase method (Au400 automatic analyzer, Olympus, Japan). Serum triglycerides (TG), total cholesterol (TC), low-density lipoprotein cholesterol (LDL-C), and high-density lipoprotein cholesterol (HDL-C) were determined by Mindray's kit and biochemical detector (BS-180, Mindray, Shenzhen, China). The glycated hemoglobin (HbA1c) assay was measured by Bio Rad reagents. Serum TSH, TPOAb, and TgAb levels were determined by the electrochemical luminescence immunoassay using the Cobas 601 analyzer (Roche Diagnostic, Switzerland). Urine iodine concentration (UIC) was examined using an inductively coupled plasma mass spectrometry (ICP-MS) (Agilent 7700x, Agilent Technologies, USA).

All participants underwent thyroid ultrasonography by qualified medical staffs using a portable instrument (LOGIQ 100 PRO, GE, Milwaukee, WI, USA with 7.5 MHz linear transducers).

### Defining Variables and Diagnostic Criteria

The normal reference ranges of serum TSH, TPOAb and TgAb were 0.27–4.20 mIU/L, ≤ 34 IU/mL, and ≤ 115 IU/mL, respectively, as provided by the manufacturer (Roche Diagnostic, Switzerland). Euthyroidism was determined based on normal serum TSH level. Serum thyroxine concentration was detected only in those subjects with abnormal TSH levels, who all had been excluded from this current study. Abdominal obesity was defined as male waist circumference ≥ 90 cm and female ≥ 85 cm (9). Hypertension was defined as receiving antihypertensive treatment or the systolic pressure ≥ 140 mmHg and/or the diastolic pressure ≥ 90 mmHg as average of two measurements. Diabetes was defined as having been diagnosed with diabetes or fasting BG (FBG) ≥ 7.0 mmol/L and/or OGTT 2 h plasma glucose (2 hPG) ≥ 11.1 mmol/L and/or HbA1c ≥ 6.5%. In non-diabetic patients, impaired fasting glucose (IFG) was defined as 5.6 ≤ FBG <7.0 mmol/L and 2 hPG < 7.8 mmol/L, while impaired glucose tolerance (IGT) was defined as FBG < 7.0 mmol/L and 7.8 ≤ 2 hPG < 11.1 mmol/L. Pre-diabetes was defined as 5.6 ≤ FBG < 7.0 mmol/L or 7.8 ≤ 2hPG < 11.1 mmol/L or 5.7% ≤ HBA1c < 6.5% (10). In terms of dyslipidemia, hypertriglyceridemia was defined as TG ≥ 1.7 mmol/L, hypercholesterolemia as TC ≥ 5.2 mmol/L, hyper-low density lipoprotein cholesterolemia as LDL-C ≥ 3.4 mmol/L, and hypo-high density lipoprotein cholesterolemia as HDL –C < 1.0 mmol/L in men and < 1.3 mmol/L in women.

### Statistical Analysis

Data are expressed as means ± standard deviation (SD), median (25–75th percentiles) or as numbers (percentages). Both statistical analyses and 1:2 random sampling were performed using IBM SPSS software, version 22.0 (IBM Corporation, Armonk, NY, USA). Analysis of variance (ANOVA) was adopted for comparisons between groups and the Bonferroni test was used for *post-hoc* analysis when continuous variables that conformed to the normal distribution were analyzed. Kruskal–Wallis test was used for comparison between groups and Mann–Whitney *U*-test was used for pairwise comparison when continuous variables that did not conform to the normal distribution were analyzed. Chi-square test was used for the comparison of those categorical variables between groups. The direct relationships between the positive expressions of thyroid autoantibodies and the prevalence of metabolic disorders were further analyzed through binary logistic regression, and reported as OR values (95% confidence interval, 95% CI). Correlation analysis of serum thyroid autoantibody titers and metabolic disorder prevalence was performed using a trend test. Statistical significance was considered when *P* < 0.05 or adjusted cutoff value due to the multiple comparisons in chi-square analysis.

## Results

### General Features of the Subjects in This Study

After exclusion and random stratification sampling, a total of 17,964 euthyroid subjects were included in the study, including 5,802 men, and 12,162 women. They were divided into TPOAb^−^TgAb^−^, TPOAb^+^TgAb^−^, TPOAb^−^TgAb^+^, and TPOAb^+^TgAb^+^ groups (Tables 1, 2). Among the males, the proportion of subjects with college education and above was markedly lower in the TPOAb^+^TgAb^−^ group than in the TPOAb^−^TgAb^−^ group, and the proportion of smokers was also lower in the TPOAb^−^TgAb^+^ group. There was a higher ratio of family history of thyroid disease in the TPOAb^+^TgAb^+^ group than in the TPOAb^−^TgAb^−^ group. Serum TSH level was higher in both TPOAb^−^TgAb^+^ and TPOAb^+^TgAb^+^ groups as compared with that of TPOAb^−^TgAb^−^ group, although their TSH levels were all under the normal range. The prevalence of goiter was significantly higher in both male and female TPOAb^+^TgAb^+^ patients than in TPOAb^−^TgAb^−^ subjects. The proportion of thyroid nodule patients in TPOAb^−^TgAb^+^ males was also markedly increased as compared with that of TPOAb^−^TgAb^−^ subjects.

**Table 1:** Characteristics of male subjects with differential expressions of serum TPOAb and TgAb.

	**TPOAb^**−**^TgAb^**−**^** **(*n* = 4,000)**	**TPOAb^**+**^TgAb^**−**^** **(*n* = 800)**	**TPOAb^**−**^TgAb^**+**^** **(*n* = 491)**	**TPOAb^**+**^TgAb^**+**^** **(*n* = 511)**
Age, years	46 (34–57)	47 (35–58)	45 (33–58)	45 (34–57)
Urban inhabitant	2,074 (51.8)	390 (48.8)	282 (57.4)	275 (53.8)
Han nationality	3,678 (92.0)	717 (89.6)	438 (89.2)	469 (91.8)
College and above education	1,228 (30.7)	207 (25.9)^#^	173 (35.2)	173 (33.9)
Income per year ≥ 30,000 yuan	2,430 (60.8)	457 (57.1)	290 (59.1)	298 (58.3)
Smoking	2,132 (53.3)	446 (55.8)	213 (43.4)^#^	248 (48.5)
With family history of thyroid disease	165 (4.2)	44 (5.5)	27 (5.6)	33 (6.5)^#^
Consumption of iodized salt	3,822 (95.6)	772 (96.5)	469 (95.5)	485 (94.9)
UIC, μg/L				
<100	605 (15.1)	140 (17.5)	96 (19.6)	94 (18.4)
100–199	1,628 (40.7)	331 (41.4)	199 (40.5)	193 (37.8)
200–299	1,037 (25.9)	202 (25.3)	117 (23.8)	131 (25.6)
≥300	730 (18.3)	127 (15.9)	79 (16.1)	93 (18.2)
TSH, mIU/L	2.12 ± 0.86	2.15 ± 0.89	2.32 ± 0.92^*^	2.44 ± 0.90^*^
Goiter	28 (0.7)	6 (0.8)	5 (1.0)	10 (2.0)^#^
Thyroid nodule	669 (16.7)	147 (18.4)	112 (22.8)^#^	87 (17.0)
BMI, kg/m^2^	24.77 ± 3.58	24.55 ± 3.70	24.76 ± 3.60	25.02 ± 3.62
WC, cm	87.46 ± 10.18	87.02 ± 10.20	87.51 ± 10.22	87.49 ± 11.11
SBP, mmHg	130.45 ± 18.31	130.52 ± 19.80	129.6 ± 18.57	130.21 ± 17.85
DBP, mmHg	81.03 ± 12.62	81.57 ± 13.78	80.03 ± 12.10	82.18 ± 14.47
FPG, mmol/L	5.54 ± 1.59	5.63 ± 1.90	5.46 ± 1.35	5.48 ± 1.41
2 hPG, mmol/L	6.71 ± 3.18	6.87 ± 3.34	6.63 ± 2.97	6.75 ± 2.96
HbA1c, %	5.61 ± 1.21	5.70 ± 1.27	5.62 ± 1.15	5.63 ± 1.16
TG, mmol/L	1.36 (0.94–2.08)	1.40 (0.95–2.10)	1.34 (0.87–1.98)	1.41 (0.96–2.05)
TC, mmol/L	4.78 ± 1.08	4.87 ± 1.18	4.68 ± 1.07	4.80 ± 0.99
LDL-C, mmol/L	2.92 ± 0.91	2.98 ± 1.05	2.89 ± 0.87	2.96 ± 0.89
HDL-C, mmol/L	1.39 ± 0.53	1.41 ± 0.51	1.42 ± 0.58	1.39 ± 0.47

*Data are presented as the mean ± standard deviation for continuous variables or as n (%) for categorical variables*.

*Age and TG are presented as median (P_25_-P_75_)*.

* *P < 0.05 as compared with that of TPOAb^−^TgAb^−^ group*.

# *P < adjusted cutoff value of P due to the multiple comparisons for statistical significance (0.05/3 = 0.017) in chi-square test*.

**Table 2:** Characteristics of female subjects with differential expressions of serum TPOAb and TgAb.

	**TPOAb^**−**^TgAb^**−**^** **(*n* = 8,000)**	**TPOAb^**+**^TgAb^**−**^** **(*n* = 1,202)**	**TPOAb^**−**^TgAb^**+**^** **(*n* = 1,588)**	**TPOAb^**+**^TgAb^**+**^** **(*n* = 1,372)**
Age, years	45 (34–56)	47 (37–57)^*^	45 (32–55)	44 (33–54)
Urban inhabitant	4,151 (51.9)	598 (49.8)	847 (53.3)	706 (51.5)
Han nationality	7,129 (89.1)	1,065 (88.6)	1,421 (89.5)	1,257 (91.6)^#^
College and above education	2,218 (27.7)	275 (22.9)^#^	458 (28.8)	389 (28.4)
Income per year ≥ 30,000 yuan	4,114 (51.4)	598 (49.8)	822 (51.8)	715 (52.1)
Smoking	275 (3.4)	50 (4.2)	37 (2.3)	44 (3.2)
With family history of thyroid disease	402 (5.1)	92 (7.7)^#^	96 (6.1)	120 (8.8)^#^
Post menopause	2,960 (37.0)	506 (42.1)^#^	563 (35.5)	483 (35.2)
Consumption of iodized salt	7,633 (95.4)	1,149 (95.6)	1,521 (95.8)	1,306 (95.2)
UIC, μg/L				
<100	1,611 (20.1)	265 (22.0)^#^	337 (21.2)	298 (21.7)
100–199	3,187 (39.8)	522 (43.4)^#^	619 (39.0)	545 (39.7)
200–299	1,779 (22.2)	235 (19.6)^#^	335 (21.1)	288 (21.0)
≥300	1,423 (17.8)	180 (15.0)^#^	288 (21.0)	241 (17.6)
TSH, mIU/L	2.24 ± 0.89	2.35 ± 0.95^*^	2.36 ± 0.93^*^	2.56 ± 0.95^*^
Goiter	79 (1.0)	20 (1.7)	22 (1.4)	49 (3.6)^#^
Thyroid nodule	1,947 (24.3)	313 (26.0)	345 (21.7)	313 (22.8)
BMI, kg/m^2^	23.57 ± 3.73	23.71 ± 3.62	23.52 ± 3.70	23.71 ± 3.65
WC, cm	80.25 ± 10.39	80.83 ± 10.10	80.38 ± 10.27	80.23 ± 10.47
SBP, mmHg	123.34 ± 20.02	125.16 ± 20.58	122.44 ± 19.74	122.21 ± 19.68
DBP, mmHg	76.34 ± 11.71	76.98 ± 12.30	76.01 ± 11.38	76.17 ± 12.58
FPG, mmol/L	5.34 ± 1.35	5.4 ± 1.45	5.24 ± 1.15^*^	5.28 ± 1.01
2 hPG, mmol/L	6.64 ± 2.69	6.77 ± 2.70	6.47 ± 2.48	6.46 ± 2.37
HbA1c, %	5.53 ± 1.10	5.60 ± 1.06	5.45 ± 0.98	5.49 ± 0.94
TG, mmol/L	1.10 (0.77–1.64)	1.09 (0.78–1.63)	1.09 (0.76–1.59)	1.06 (0.76–1.61)
TC, mmol/L	4.75 ± 1.10	4.84 ± 1.14	4.71 ± 1.12	4.72 ± 1.07
LDL-C, mmol/L	2.78 ± 0.91	2.85 ± 0.96	2.82 ± 1.03	2.79 ± 0.96
HDL-C, mmol/L	1.6 ± 0.58	1.62 ± 0.63	1.64 ± 0.62^*^	1.58 ± 0.53

*Data are presented as the mean ± standard deviation for continuous variables or as n (%) for categorical variables*.

*Age and TG are presented as median (P_25_-P_75_)*.

* *P < 0.05 as compared with that of TPOAb^−^TgAb^−^ group*.

# *P < adjusted cut-off value of P due to the multiple comparisons for statistical significance (0.05/3 = 0.017) in chi-square test*.

Among the female subjects, both the average age of the TPOAb^+^TgAb^−^ group and the proportion of menopausal women were significantly higher as compared with those of the TPOAb^−^TgAb^−^ group. The percentage of females with college education and above was pronouncedly lower in the TPOAb^+^TgAb^−^ group, and the proportion of Han people was significantly higher in the TPOAb^+^TgAb^+^ group than in the TPOAb^−^TgAb^−^ group. There was a higher ratio of family history of thyroid diseases in both TPOAb^+^TgAb^−^ and TPOAb^+^TgAb^+^ groups. Serum TSH level was markedly higher in TPOAb^+^TgAb^−^, TPOAb^−^TgAb^+^, and TPOAb^+^TgAb^+^ groups, although their serum TSH concentrations were all under the normal range. The iodine nutritional status of the TPOAb^+^TgAb^−^ group was different from that of the TPOAb^−^TgAb^−^ group. The proportion of the subjects with UIC > 200 μg/L (i.e., more than adequate and excess iodine intake) was significantly lower in the former group (*P* < 0.01).

### Association of Glucose and Lipid Levels to the Positivity of Thyroid Autoantibodies in the Serum

Both the actual blood levels of glucose and lipid and the incidence of related metabolic disorders (e.g., hyperglycemia and dyslipidemia) were analyzed based on the differential expression patterns of TPOAb and TgAb in the serum, and all the comparisons were made with that of the TPOAb^−^TgAb^−^ group (Tables 1–3). Due to the multiple comparisons in chi-square analysis for statistical significance, the cutoff *P*-value was accordingly adjusted to 0.017 (0.05/3) rather than 0.05.

**Table 3:** Percentage of subjects with hyperglycemia and dyslipidemia among those with differential expressions of serum TPOAb and TgAb.

	**TPOAb^**−**^TgAb^**−**^**	**TPOAb^**+**^TgAb^**−**^**	**TPOAb^**−**^TgAb^**+**^**	**TPOAb^**+**^TgAb^**+**^**
**MEN**				
N (%)	4,000 (68.9)	800 (13.8)	491 (8.5)	511 (8.8)
Central obesity	1,719 (43.0)	329 (41.1)	213 (43.4)	222 (43.4)
Hypertension	1,596 (39.9)	335 (41.9)	180 (36.7)	202 (39.5)
**HYPERGLYCEMIA**				
IFG	553 (13.8)	104 (13.0)	50 (10.2)^a^	66 (12.9)
IGT	282 (7.0)	61 (7.6)	37 (7.5)	41 (8.0)
Pre-diabetes	1,601 (40.0)	319 (39.9)	195 (39.7)	203 (39.7)
Diabetes	599 (15.0)	134 (16.8)	84 (17.1)	75 (14.7)
**DYSLIPIDEMIA**				
High TG	1,432 (35.8)	297 (37.1)	170 (34.6)	188 (36.8)
High TC	1,273 (31.8)	266 (33.3)	164 (33.4)	162 (31.7)
High LDL-C	1,007 (25.2)	215 (26.9)	124 (25.3)	136 (26.6)
Low HDL-C	602 (15.0)	126 (15.8)	76 (15.5)	77 (15.1)
**WOMEN**				
N (%)	8,000 (65.8)	1,202 (9.9)	1,588 (12.0)	1,372 (11.3)
Central obesity	2,616 (32.7)	388 (32.3)	502 (31.6)	443 (32.3)
Hypertension	2,214 (27.7)	361 (30.0)	423 (26.6)	359 (26.2)
**HYPERGLYCEMIA**				
IFG	742 (9.3)	107 (8.9)	149 (9.4)	120 (8.7)
IGT	685 (8.6)	112 (9.3)	111 (7.0)^b^	108 (7.9)
Pre-diabetes	2,927 (36.6)	476 (39.6)	554 (34.9)	499 (36.4)
Diabetes	958 (12.0)	142 (11.8)	169 (10.6)	147 (10.7)
**DYSLIPIDEMIA**				
High TG	1,903 (23.8)	276 (23.0)	341 (21.5)^c^	311 (22.7)
High TC	2,505 (31.3)	414 (34.4)^d^	490 (30.9)	412 (30.0)
High LDL-C	1,676 (21.0)	275 (22.9)	356 (22.4)	298 (21.7)
Low HDL-C	2,195 (27.4)	310 (25.8)	420 (26.4)	383 (27.9)

*Data are presented as n (%)*.

a *P = 0.026*,

b *P = 0.038*,

c *P = 0.047*,

d *P = 0.03 as compared with that of TPOAb^−^TgAb^−^ group, and none of them was less than the adjusted cutoff value of P due to the multiple comparisons for statistical significance (0.05/3 = 0.017) in chi-square test*.

Among the males, the incidence of IFG in the TPOAb^−^TgAb^+^ group showed a decreased trend (0.017 < *P* = 0.026 < 0.05) as compared with that of TPOAb^−^TgAb^−^ group. Among the females, the incidence of hypercholesterolemia exhibited an increased tendency in the TPOAb^+^TgAb^−^ group (0.017 < *P* = 0.03 < 0.05). There were significantly lower FBG (*P* < 0.05) and higher HDL-C levels (*P* < 0.05) as well as tendencies toward decreased incidences of IGT (0.017 < *P* = 0.038 < 0.05) and hypertriglyceridemia (0.017 < *P* = 0.047 < 0.05) in the TPOAb^−^TgAb^+^ group. Those *P*-values between 0.017 and 0.05 suggested no statistical significance.

### Confirmation of Independent Effects of Thyroid Autoantibodies Through Binary Logistic Regression Analysis

To further investigate whether there were independent effects of thyroid autoantibodies on metabolic disorders related to glucose and lipids, a binary logistic regression analysis was performed. The TPOAb^−^TgAb^−^ group was used as the reference. We calculated the OR value of the occurrence of hyperglycemia and dyslipidemia in TPOAb^+^TgAb^−^, TPOAb^−^TgAb^+^, and TPOAb^+^TgAb^+^ groups for both males and females. The confounding factors adjusted in Model 1–3 of binary logistic regression consisted of age, nationality, location, education level, family income, smoking, family history of thyroid diseases, menopausal status (only in females), UIC, BMI (not included for the analysis of central obesity), and TSH, respectively (Table 4).

**Table 4:** Odds ratio of hyperglycemia and dyslipidemia among the euthyroid population with differential expressions of serum TPOAb and TgAb.

	**Model 1**			**Model 2**			**Model 3**		
	**TPOAb^**+**^TgAb^**−**^**	**TPOAb^**−**^TgAb^**+**^**	**TPOAb^**+**^TgAb^**+**^**	**TPOAb^**+**^TgAb^**−**^**	**TPOAb^**−**^TgAb^**+**^**	**TPOAb^**+**^TgAb^**+**^**	**TPOAb^**+**^TgAb^**−**^**	**TPOAb^**−**^TgAb^**+**^**	**TPOAb^**+**^TgAb^**+**^**
**MEN**									
Central obesity	0.919 (0.787–1.073)	1.022 (0.845–1.237)	1.025 (0.850–1.236)	0.934 (0.798–1.092)	1.035 (0.852–1.256)	1.050 (0.870–1.268)	0.924 (0.790–1.082)	1.002 (0.825–1.218)	1.015 (0.839–1.228)
Hypertension	1.070 (0.909–1.260)	0.868 (0.706–1.067)	1.003 (0.822–1.223)	1.087 (0.922–1.282)	0.902 (0.732–1.112)	1.003 (0.821–1.226)	1.085 (0.915–1.287)	0.840 (0.677–1.043)	0.934 (0.758–1.150)
**HYPERGLYCEMIA**									
IFG	0.925 (0.739–1.159)	0.708 (0.521–0.962)^*^	0.929 (0.706–1.222)	0.926 (0.739–1.161)	0.697 (0.508–0.955)^*^	0.943 (0.716–1.242)	0.926 (0.738–1.161)	0.691 (0.503–0.949)^*^	0.935 (0.708–1.234)
IGT	1.078 (0.807–1.439)	1.080 (0.755–1.544)	1.163 (0.825–1.638)	1.068 (0.797–1.429)	1.056 (0.734–1.519)	1.157 (0.821–1.631)	1.064 (0.794–1.426)	1.055 (0.732–1.520)	1.195 (0.845–1.690)
Pre-diabetes	0.982 (0.839–1.150)	0.994 (0.818–1.207)	0.996 (0.823–1.205)	0.987 (0.843–1.156)	0.986 (0.809–1.201)	0.996 (0.822–1.206)	0.990 (0.845–1.161)	0.987 (0.809–1.204)	1.004 (0.827–1.218)
Diabetes	1.128 (0.913–1.394)	1.190 (0.918–1.543)	1.000 (0.765–1.307)	1.147 (0.927–1.419)	1.218 (0.937–1.584)	0.981 (0.748–1.286)	1.134 (0.914–1.407)	1.172 (0.898–1.530)	0.933 (0.708–1.231)
**DYSLIPIDEMIA**									
High TG	1.058 (0.904–1.238)	0.950 (0.781–1.157)	1.045 (0.863–1.265)	1.061 (0.905–1.244)	1.011 (0.828–1.235)	1.081 (0.891–1.311)	1.061 (0.898–1.254)	0.971 (0.788–1.197)	1.013 (0.828–1.240)
High TC	1.058 (0.899–1.246)	1.082 (0.885–1.323)	1.002 (0.821–1.223)	1.054 (0.895–1.242)	1.107 (0.903–1.358)	1.009 (0.825–1.234)	1.043 (0.883–1.233)	1.085 (0.882–1.335)	1.006 (0.820–1.236)
High LDL-C	1.086 (0.914–1.291)	1.008 (0.812–1.252)	1.084 (0.879–1.337)	1.083 (0.910–1.289)	1.004 (0.806–1.252)	1.090 (0.882–1.346)	1.076 (0.901–1.285)	0.988 (0.789–1.238)	1.103 (0.888–1.369)
Low HDL-C	1.086 (0.914–1.291)	1.008 (0.812–1.252)	1.084 (0.879–1.337)	1.065 (0.863–1.316)	1.089 (0.837–1.416)	1.026 (0.792–1.328)	1.064 (0.858–1.321)	1.074 (0.820–1.405)	0.968 (0.742–1.263)
**WOMEN**									
Central obesity	0.894 (0.780–1.025)	0.975 (0.863–1.102)	1.030 (0.905–1.172)	0.888 (0.773–1.019)	0.989 (0.874–1.119)	1.043 (0.915–1.190)	0.876 (0.763–1.007)	0.984 (0.870–1.114)	1.010 (0.884–1.154)
Hypertension	1.002 (0.864–1.163)	0.982 (0.857–1.126)	0.983 (0.849–1.138)	0.991 (0.853–1.121)	0.977 (0.851–1.121)	0.975 (0.841–1.130)	1.002 (0.860–1.168)	0.960 (0.833–1.106)	0.928 (0.796–1.081)
**HYPERGLYCEMIA**									
IFG	0.922 (0.745–1.141)	1.026 (0.852–1.235)	0.955 (0.780–1.170)	0.895 (0.721–1.112)	1.025 (0.850–1.235)	0.945 (0.771–1.160)	0.907 (0.731–1.127)	1.028 (0.853–1.240)	0.967 (0.787–1.188)
IGT	1.051 (0.851–1.298)	0.813 (0.660–1.002)	0.933 (0.754–1.154)	1.062 (0.859–1.312)	0.813 (0.659–1.002)	0.913 (0.737–1.132)	1.079 (0.873–1.334)	0.821 (0.665–1.013)	0.936 (0.753–1.163)
Pre-diabetes	1.077 (0.947–1.223)	0.944 (0.840–1.060)	1.024 (0.906–1.157)	1.078 (0.948–1.226)	0.947 (0.843–1.064)	1.013 (0.895–1.146)	1.086 (0.954–1.236)	0.946 (0.841–1.063)	1.014 (0.895–1.149)
Diabetes	0.882 (0.724–1.074)	0.900 (0.750–1.080)	0.920 (0.758–1.117)	0.886 (0.726–1.081)	0.898 (0.747–1.079)	0.924 (0.760–1.123)	0.891 (0.729–1.090)	0.883 (0.733–1.064)	0.899 (0.737–1.096)
**DYSLIPIDEMIA**									
High TG	0.890 (0.768–1.032)	0.890 (0.778–1.018)	0.971 (0.843–1.117)	0.886 (0.763–1.028)	0.886 (0.774–1.014)	0.968 (0.840–1.115)	0.884 (0.759–1.030)	0.859 (0.748–0.987)^*^	0.904 (0.781–1.047)
High TC	1.068 (0.933–1.223)	1.007 (0.890–1.139)	0.983 (0.862–1.122)	1.058 (0.922–1.213)	1.002 (0.885–1.135)	0.957 (0.838–1.094)	1.058 (0.922–1.214)	1.011 (0.892–1.146)	0.961 (0.839–1.100)
High LDL-C	1.042 (0.896–1.211)	1.129 (0.987–1.292)	1.098 (0.950–1.269)	1.027 (0.882–1.196)	1.141 (0.997–1.307)	1.099 (0.950–1.272)	1.037 (0.889–1.208)	1.147 (1.000–1.316)	1.094 (0.943–1.270)
Low HDL-C	0.905 (0.788–1.039)	0.956 (0.846–1.080)	1.033 (0.909–1.174)	0.911 (0.792–1.047)	0.949 (0.839–1.073)	1.037 (0.911–1.179)	0.912 (0.791–1.051)	0.927 (0.817–1.051)	0.972 (0.851–1.110)

*Model 1. Adjusted for age*.

*Model 2. Adjusted for age, location, nationality, education level, family income, smoking, family history of thyroid disease and menopausal status (only in females)*.

Model 3. Adjusted for age, location, nationality, education level, family income, smoking, family history of thyroid disease, menopausal status (only in females), BMI (but not included for central obesity in the regression model), TSH, UIC, thyroid nodule, and goiter;

* *P < 0.05*.

There were similar findings from Model 1, Model 2, and Model 3 analyses (Table 4), which indicated that TgAb single positivity was an independent protective factor associated with lower risk of IFG in males [OR (95% CI) in Model 1: 0.708 (0.521–0.962); Model 2: 0.697 (0.508–0.955); Model 3: 0.691 (0.503–0.949)]. After further adjustment by UIC, BMI, TSH, thyroid nodule and goiter besides age, nationality, location, education level, family income, smoking, family history of thyroid diseases, menopausal status in Model 3, TgAb single positivity was shown to be an independent protective factor related to lower risk of hypertriglyceridemia in females [OR (95% CI) in Model 3: 0.859 (0.748–0.987)] (Table 4). The relationship between TgAb single positivity and IFG or hypertriglyceridemia was not dependent on serum TSH level, although the latter was slightly higher in the TPOAb^−^TgAb^+^ group than in the TPOAb^−^TgAb^−^ group.

### The Relationship Between the Prevalence of IFG and Hypertriglyceridemia and Serum TgAb Level

The associations between serum TgAb level and the proportions of those people with IFG and hypertriglyceridemia were further investigated. They were also conducted after stratification by either serum TSH concentration (≤ 2.50 and > 2.50 mIU/L) or BMI (< 28.0 and ≥ 28.0 kg/m^2^) since thyroid functions are well-known to have important effects on the metabolism of glucose and lipids, and consequentially, obesity. Per NCAB recommendation, the upper normal limit of serum TSH is 2.50 mIU/L (11). The cutoff value for the diagnosis of obesity in Chinese adults is 28.0 kg/m^2^ (12). The lower detection limit of TgAb was 10.0 IU/mL as provided by the manufacturer. In the TgAb^+^ males and females, average serum TgAb level was 297.0 (187.0–465.9) and 315.1 (198.9–466.5) IU/mL, respectively. Serum TgAb was divided into 4 levels (≤ 40.0, 40.0–115.0, 115.0–350.0, > 350.0 IU/mL) for a trend test of concentration-dependence.

**Figure 2:**
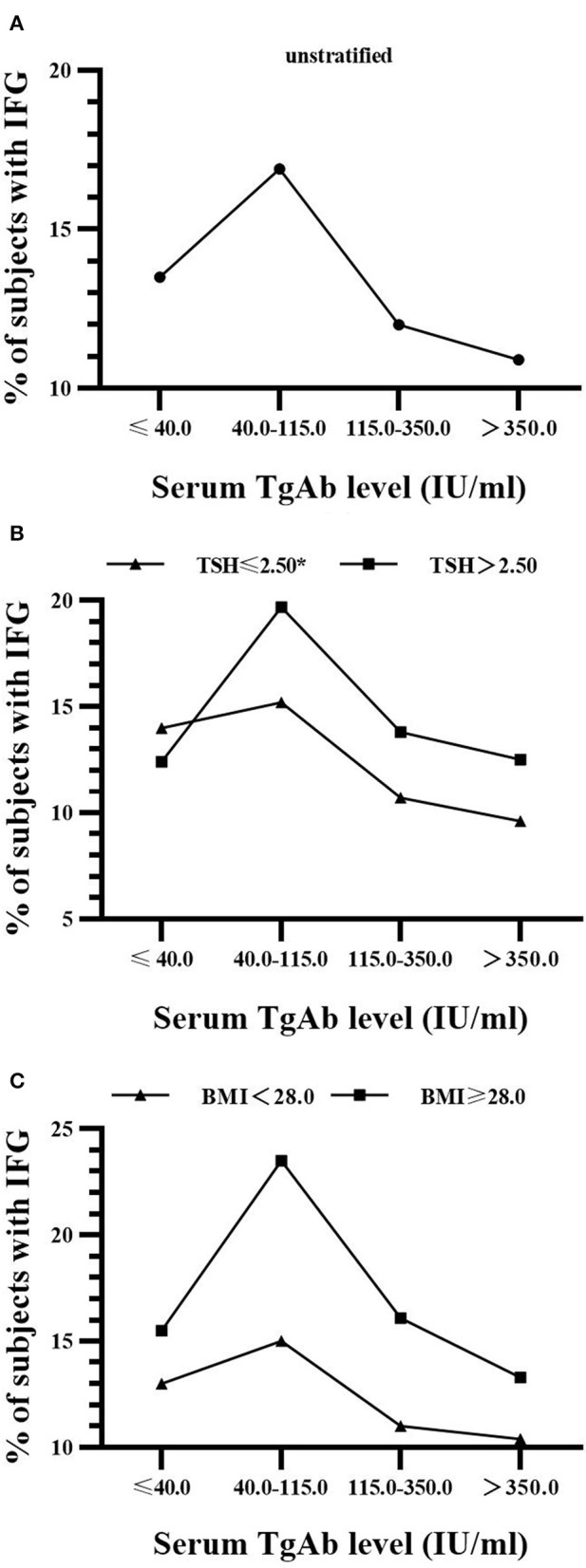
Serum TgAb level and the percentage of subjects with IFG in men **(A)** Serum TgAb level and the percentage of subjects with IFG in all males. *P* for trend = 0.162 **(B)** Serum TgAb level and the percentage of subjects with IFG in males after stratified by TSH. TSH ≤ 2.5 mIU/L, *P for trend = 0.024; TSH > 2.50 mIU/L, *P* for trend = 0.548 **(C)** Serum TgAb level and the percentage of subjects with IFG in males after stratified by BMI. BMI < 28.0 kg/m^2^, *P* for trend = 0.109; BMI ≥ 28.0 kg/m^2^, *P* for trend = 0.962.

**Figure 3:**
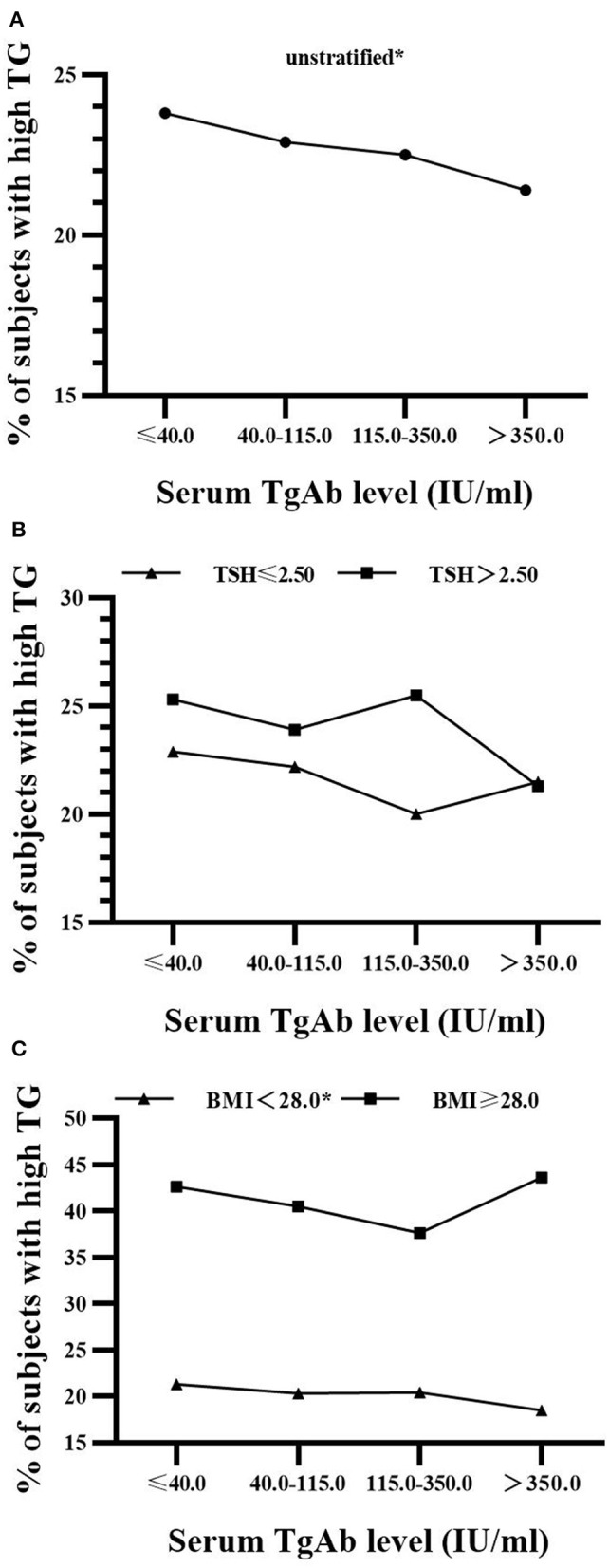
Serum TgAb level and the percentage of subjects with hypertriglyceridemia in women **(A)** Serum TgAb level and the percentage of subjects with hypertriglyceridemia (high TG) in all females. *P for trend = 0.04 **(B)** Serum TgAb level and the percentage of subjects with high TG in females after stratified by TSH. TSH ≤ 2.5 mIU/L, *P* for trend = 0.095; TSH > 2.50 mIU/L, *P* for trend = 0.101 **(C)** Serum TgAb level and the percentage of subjects with high TG in females after stratified by BMI. BMI < 28.0 kg/m^2^, *P for trend = 0.033; BMI ≥ 28.0 kg/m^2^, *P* for trend = 0.569.

As shown in Figure 2, there was a significant decrease (14.0, 15.2, 10.7, 9.6%; *P* for trend = 0.024) in the prevalence of IFG with the rise of serum TgAb level in euthyroid men with TSH ≤ 2.5 mIU/L. However, its reduction was not statistically significant in males with TSH > 2.5 mIU/L regardless of BMI (Figure 2). In addition, the proportion of hypertriglyceridemia patients was markedly decreased (21.3, 20.3, 20.4, 18.5%; *P* for trend = 0.033) with the rise of serum TgAb level in the non-obese women (BMI < 28.0 kg/m^2^, Figure 3). It did not show a statistically significant change when female BMI was ≥ 28 kg/m^2^ (obese) or only stratified by serum TSH (Figure 3). Consistent with the results from logistic regression analysis, the findings above further suggest a potential, titer-dependent and independent role of TgAb in lowering the incidence of male IFG and female hypertriglyceridemia.

## Discussion

The presence of thyroid autoantibodies in the serum usually indicates the occurrence of thyroid autoimmune responses, which may result in abnormal thyroid functions (1). Our previous studies have reported that the positive rates of TPOAb and TgAb in the serum were as high as 11.5 and 12.6% in Chinese general population, respectively (3). The metabolic disorders related to glucose and lipid are the most common endocrine diseases. Many studies have focused on the effects of thyroid hormones on glucose and lipid metabolism (13). However, few studies have explored the relationship between the positivity of thyroid autoantibodies and those metabolic disorders; these studies have been summarized in Table 5 (6–8, 14–21). In a small sample study, TPOAb expression was not related to the presence of dyslipidemia in the sub-clinically hypothyroid subjects (21). Kang et al. found that the differences in serum lipid levels between TPOAb^+^ and TPOAb^−^ subjects were dependent on serum TSH concentration (16). Both studies suggest that TSH may be one of the main confounding factors in the analysis of the influences of thyroid autoantibodies on lipid metabolism. A study of euthyroid Iranians showed that none of metabolic syndrome components was expressed in association with TPOAb positivity (17). However, none of the above studies had investigated serum TgAb. In Portuguese METabolic Syndrome study, the positivity of serum TPOAb was negatively correlated with hypertriglyceridemia (OR: 0.321, 95% CI: 0.124–0.836), and TgAb was not associated with the components of metabolic syndrome (8). However, stratification analysis by gender was not performed and subjects with thyroid dysfunctions were not excluded in that study, which might explain the difference between their findings and ours. Another investigation of euthyroid adults in East China recently reported that TPOAb and/or TgAb expressions were positively correlated with HbA1c, obesity and dyslipidemia, especially in females (6). In that survey, the individual roles of TPOAb and TgAb expressions were not investigated. In a recent meta-analysis of obesity and thyroid autoimmunity, obesity was correlated with positive serum TPOAb expression (RR = 1.93, 95% CI: 1.31–2.85), but not with TgAb (22). In that study, the subjects with thyroid dysfunctions were not excluded and the association of thyroid autoimmunity with either hyperglycemia or dyslipidemia was not investigated (22). Thus, there is not only a lack of consistent findings in the relationship between thyroid autoantibody expression and the occurrence of metabolic disorders, but also of the understanding of the respective roles of TPOAb and TgAb, which need to be further investigated.

**Table 5:** Summary of the studies related to TPOAb, TgAb, and metabolic disorders of glucose and lipids.

	**References**	**Subjects**	**Grouping for analysis**	**Findings**
1	Raposo et al. (8)	① 281 females and 205 males ② ≥18 years old ③ with euthyroidism, hyperthyroidism or hypothyroidism	① grouping as TPOAb+, TgAb+, TPOAb+, and/or TgAb+ (TPOAb+/TgAb+), both TPOAb+, and TgAb+ (TPOAb+ TgAb+) ② not stratified by gender	① TPOAb positivity was negatively related to the prevalence of metabolic syndrome and its TG component while TgAb had no association. ② in logistic regression model, only age and sex were adjusted.
2	Chen et al. (6)	① 5,134 females and 3,948 males from East China ② ≥18 years old ③ with euthyroidism	① grouping as TPOAb+/TgAb+, both TPOAb-, and TgAb- (TPOAb-TgAb-) ② stratified by gender	① TPOAb+/TgAb+ was related to an increased risk for central obesity, hyperlipidemia, and metabolic syndrome in females, but not in males. ② in logistic regression model, age, smoking history, TSH, BMI, and menopausal status in women were adjusted. ③ linear regression showed that B was −0.021 between TPOAb+/TgAb+ and FPG level (*P* = 0.055) in men while B was 0.032 between TPOAb+/TgAb+ and TG level (*P* = 0.052) in women.
3	Liu et al. (14)	① 4,134 non-obese females and 1,474 non-obese males from Beijing Chao-Yang Hospital ② averagely aged 45–46 years old ③ with euthyroidism	① grouping as TPOAb+/TgAb+ and TPOAb-TgAb- ② not stratified by gender	① blood TG and FBG levels were lower in TPOAb+/TgAb+ group (*P* = 0.054 and 0.075, respectively). ② serum TPOAb titer positively correlated with the levels of TC, TG, HOMA-IR and hsCRP in non-obese individuals, but TgAb titer did not. ③ no regression analysis was performed between TPOAb and lipids.
4	Sarfo-Kantanka et al. (15)	① 178 females and 124 males with T2DM; 181 females and 129 males as controls ② 40–80 years old ③ with euthyroidism, hyperthyroidism or hypothyroidism	① grouping as TPOAb+/TgAb+ and TPOAb-TgAb- ② not stratified by gender	① TPOAb+/TgAb+ in T2DM patients may be related to the increased risk of hypercholesterolemia and poor glycemic control. ② in logistic regression model, age, sex and thyroid functions were adjusted.
5	Kang et al. (16)	① 1,607 subjects (male: female 1:3.7) ② 35–65 years old ③ with euthyroidism	① grouping as TPOAb+ and TPOAb- (TgAb not measured) ② not stratified by gender	① TPOAb positivity was associated with the increased levels of blood TC, LDL-C and HDL-C, but not TG. ② no regression analysis was performed.
6	Amouzegar et al. (17)	① 1,172 females and 1,531 males ② ≥20 years old ③ with euthyroidism	① grouping as TPOAb+ and TPOAb- (TgAb not measured) ② stratified by gender	① no differences were observed in HOMA-IR, blood lipids, BMI, BP, or FBG between TPOAb+ and TPOAb- groups. ② no regression analysis was performed.
7	Agbaht et al. (7)	① 484 females and 64 males with obesity ② 35–51 years old ③ with euthyroidism or hypothyroidism	① grouping as obesity with and without metabolic syndrome ② TgAb not measured ③ not stratified by gender	① serum TPOAb level was not correlated with the occurrence of metabolic syndrome. ② in regression model, age, sex, TSH, HOMA-IR, BMI, family history, and smoking were adjusted.
8	Mazaheri et al. (18)	① 98 females and 14 males ② 41.8 ± 12.8 years old ③ with euthyroidism due to levothyroxine replacement therapy.	① grouping as TPOAb negative (<80 IU/ml), positive (80–1,000), highly positive (>1,000) ② TgAb not measured ③ not stratified by gender.	① those subjects with highly positive TPOAb may have higher serum insulin and lower HDL-c levels when BMI at same. ② no regression analysis was performed.
9	Topaloglu et al. (19)	① 66 premenopausal females ② 17–50 years old ③ with euthyroidism	① grouping as TPOAb+/TgAb+ and TPOAb- TgAb- ② no males included	① both serum TPOAb and TgAb levels positively correlated with blood TC, LDL-C, and negatively correlated with HDL-C, and serum TgAb was also positively associated with TG level. ② no regression analysis was performed.
10	Tamer et al. (20)	① 334 females ② premenopausal stage ③ with euthyroidism or hypothyroidism	① grouping as TPOAb+/TgAb+ and TPOAb- TgAb- ② no males included	① serum TPOAb level positively correlated with TG concentration and waist circumferences, and negatively with HDL-C; serum TgAb also positively correlated with TG and non-HDL-C concentrations. ② no regression analysis was performed.
11	Wells et al. (21)	① 130 females and 58 males ② ≥40 years old ③ with subclinical hypothyroidism	① grouping as TPOAb+ and TPOAb- (TgAb not measured) ② not stratified by gender	① TPOAb positivity may not be related to the increased blood lipid levels in the patients with subclinical hypothyroidism. ② no regression analysis was performed.
12	The current study	① 12,162 females and 5,802 males from 31 provinces of mainland China ② ≥18 years old ③ with euthyroidism	① grouping as TPOAb-TgAb-, TPOAb+TgAb-, TPOAb-TgAb+, TPOAb+TgAb+ ② stratified by gender	① serum TgAb single positivity may imply a reduced risk of IFG in men and of hypertriglyceridemia in women. ② in logistic regression model, age, nationality, location, education level, family income, smoking, family history of thyroid diseases, menopausal status (in women), urinary iodine, BMI, TSH, thyroid nodule and goiter were all adjusted.

Based on a large-scale cross-sectional survey, this study analyzed the relationship between the positivity of thyroid autoantibodies and the prevalence of metabolic disorders related to glucose and lipids. It showed that among the euthyroid population, TgAb single positivity was an independent protective factor associated with lower risks of IFG in males and of hypertriglyceridemia in females. Although there have been some related studies published (Table 5), none of them has addressed the above association after stratification by the expression patterns of thyroid autoantibodies as TPOAb^+^TgAb^−^, TPOAb^−^TgAb^+^, TPOAb^+^TgAb^+^, and TPOAb^−^TgAb^−^. We found that there were decreased trends in both FBG concentration and the proportion of individuals with IFG in TPOAb^−^TgAb^+^ males as compared with those of TPOAb^−^TgAb^−^ males. There were significantly lower FBG and higher HDL-C levels as well as trends toward decreased incidences of IGT and hypertriglyceridemia in the TPOAb^−^TgAb^+^ females when compared with those of TPOAb^−^TgAb^−^ females. Furthermore, binary logistic regression analysis showed that serum TgAb single positivity was an independent protective factor for IFG in males and for hypertriglyceridemia in females. Trend test showed that, with the increase of serum TgAb level, there were titer-dependent decreases in the prevalence of IFG among the euthyroid men (especially in those with TSH ≤ 2.5 mIU/L) and that of hypertriglyceridemia in euthyroid women (especially in non-obese women). These findings suggest the potential protective roles of TgAb are indeed independent of thyroid functions and body weight. The mechanisms related to these interesting phenomena await further investigation. In fact, the results from Liu et al. have shown that serum HDL-C level was elevated and serum TG and FBG were lower in non-obese AIT patients as compared with that of the thyroid-autoantibody-negative controls (14). However, they did not stratify by gender, and relatively less male AIT patients were included (*n* = 366), which may impact the findings.

Education, residence, annual household income, smoking, and BMI have been found with some effects on glucose and lipid metabolism (23–26). It has recently been reported that iodine intake status may also affect blood glucose and lipid levels (27). Furthermore, education level, iodine status, smoking, and obesity may affect the development of thyroid autoimmunity and the production of thyroid autoantibodies (22, 28–30). Therefore, we included all above factors into logistic regression as potentially confounding, and further analyzed whether thyroid autoantibodies were independently associated with metabolic disorders of glucose and lipid. Our results suggest that the positivity of TgAb alone in the serum may independently affect the glucose and lipid metabolism. Due to lack of related reports until now, possible mechanisms of how serum TgAb expression are associated with lower risk of male IFG and female hypertriglyceridemia are not understood. Carbonic anhydrase (CA) isozymes belong to a family of zinc-containing enzymes and are widely existent in mammals (31). A recent study has found that polymorphism of CA was related to serum TgAb level (32). When the activity of CA was decreased, the iodine uptake by the thyroid gland was increased, and this process was not affected by TSH (33). High levels of iodine in the thyroid gland can lead to an increase in Tg iodization, which may enhance the antigenicity of Tg and the production of TgAb (34). Additionally, low CA activity can reduce ${\text{HCO}}_{3}^{-}$ production in the hepatocytes, which may result in decreased availability of gluconeogenesis substrate in the liver and subsequently decrease both hepatic glucose production and FPG (35). Thus, the above findings suggest that the negative correlation between serum TgAb positive expression and the occurrence of IFG may be due to potentially abnormal CA activity. Another mechanism may be involved in the production of anti-CA autoantibodies due to epitope mimics. CA consists of at least 15 isoforms located in the cytoplasm or on the membrane (36). It has been reported that autoantibodies against CA were present in the patients with some autoimmune diseases, such as autoimmune pancreatitis, rheumatoid arthritis, and systemic lupus erythematosus (37–39). In addition, anti-CA autoantibodies have been suggested to be capable of suppressing the total CA activity and influencing its normal physiological functions (40). Previous studies on the pathogenesis of autoimmune pancreatitis have found that *Helicobacter pylori* (H. pylori) infection can induce anti-CA autoantibody production due to the highly homologous structure between bacterial α-class carbonic anhydrase and human CA-II (37). H. pylori infection is also involved in the development of AITD (41). Specifically, TgAb was significantly more prevalent in infected AITD patients than in those without infections (42). It has been found that Tg shares putative conserved structure with H. pylori proteins, while TPO only exhibits a partial linear homology and thus has less potentials for epitope mimics (42). In China, the prevalence of people infected by H. pylori is 41.5–72.3% (43). Based on these findings, we hypothesize that H. pylori infection may induce the production of both TgAb and anti-CA autoantibodies at the same time due to molecular mimicry, while the latter may inhibit hepatic CA activities and lead to lowered FBG. However, these mechanisms need to be confirmed by a direct experimental study in the future.

It has been shown that only TgAb level is elevated in the serum at the early stage of AIT, and both TPOAb and TgAb levels are increased at the late stage. The sole expression of TgAb reflects an initial immune response and the production of TPOAb is due to late immune response (44). The development of AIT is accompanied by elevated production of some cytokines. It has been found that the serum levels of IFN-γ and IL-17 were significantly increased in the mice with experimental autoimmune thyroiditis (EAT), and both IFN-γ and IL-17 contributed to the production of TgAb (45). Another study has shown that the levels of blood glucose and triglycerides were significantly elevated when the concentration of serum IFN-γ was reduced in the mice with high-fat diet as compared with that of the control group (46). These results indicate that high level of serum IFN-γ might contribute to the decreases in blood glucose and triglyceride levels. However, according to a recent report, IL-17 appeared to have no effect on glucose and lipid metabolism (47). These studies suggest that the decrease in the prevalence of IFG and hypertriglyceridemia associated with elevated serum TgAb may also result from the increased IFN-γ secretion in the pathogenesis of AIT. Of course, serum TgAb positivity has also been found at low concentrations in about 4.2% of normal subjects and some non-AITD patients, which include those with non-toxic multinodular goiter, papillary thyroid carcinoma, and subacute thyroiditis (48–50). AITD patients may or may not present with goiter or nodules (30). In this study, participants with abnormal TSH and personal history of thyroid diseases (e.g., thyroid cancer) were excluded. All the enrolled subjects underwent thyroid ultrasonography, and no typical ultrasonic changes of subacute thyroiditis were found. The prevalence of goiter was indeed significantly higher in both male and female TPOAb^+^TgAb^+^ patients than in TPOAb^−^TgAb^−^ subjects, and the proportion of thyroid nodule patients in TPOAb^−^TgAb^+^ males was also markedly increased. However, we did not focus on the associations between thyroid autoantibodies and thyroid goiter/nodules in this study, and both conditions had been adjusted as potential confounding factors in the logistic regression analysis, in which TgAb single positivity was still shown as an independent protective factor related to lower risks of IFG in males and of hypertriglyceridemia in females. In addition, these effects of TgAb exhibited titer-dependence, and it appeared to have more obvious protective roles at higher levels. The average TgAb level in the serum was obviously higher in the AITD patients than non-AITD ones (49). So, the findings above suggest autoimmunity-related mechanisms are more likely involved, which would be further explored using EAT model in our future work. Besides, the existence of serum IgG against Tg may not only enhance the clearance of circulatory Tg (51), but can also interfere with Tg detection using immunometric assays (52), which could not be solved by mass spectrometry-based Tg quantification (53). We have not measured serum Tg level in the current study, which is one of our limitations. However, to our knowledge, no direct evidence has been reported yet about the influence of Tg on the metabolism of glucose and lipids.

In addition, our results showed gender differences in the relationship between thyroid autoantibodies and glucose/lipid metabolism. We have found that serum TgAb single positivity seems to be an independent protective factor for IFG in males and for hypertriglyceridemia in females. In fact, previous studies have indicated that smoking is a risk factor for hypertriglyceridemia (26). It is likely that the protective effect of TgAb on hypertriglyceridemia was much weaker than the risk from smoking. Consequently, the protective effect of TgAb on hypertriglyceridemia was not apparent in men as shown in women since a higher proportion of smokers in the men was found than that of the women in our study. Epidemiological studies in China have found that the prevalence of diabetes in premenopausal women was significantly lower than that of men at the same age (54). Animal experiments have shown that estrogens can promote insulin secretion, reduce insulin resistance, and have a protective effect on diabetes (55). In this study, pre-menopausal women accounted for the majority (62.9%) in the females. We hypothesize that the protective effect of estrogens on female blood glucose may be stronger than that of TgAb, and the latter effect may not overtly appear in women.

The current study has been performed with large sample data from an epidemiological survey covering 31 provinces in China, and is more representative of the general Chinese population when compared with previous studies (Table 5). Most of the prior studies focused on TPOAb but not TgAb. However, the impacts of TPOAb and TgAb may be different according to the stratified analysis in this study. The limitation of this study is that it is a cross-sectional study, and the causal relationship cannot be confirmed. Additionally, there is a lack of basic experiments to investigate the mechanisms for the effects of thyroid autoantibodies on glucose and lipid metabolism. We have been establishing an EAT mouse model to study the related mechanisms of the interesting relationship between serum TgAb single positivity and the lowered risks of IFG in males and hypertriglyceridemia in females.

## Conclusion

Serum TgAb single positivity may imply a reduced risk of IFG in euthyroid men and of hypertriglyceridemia in euthyroid women. The mechanisms related to these interesting phenomena await further investigation through basic research work.

## Data Availability Statement

The datasets generated for this study are available on request to the corresponding author.

## Ethics Statement

The studies involving human participants were reviewed and approved by Medical Ethics Committee of China Medical University. The patients/participants provided their written informed consent to participate in this study.

## Author Contributions

JL, ZS, and WT conceived and designed the study and interpreted the results. JinjZ, YG, YoL, and DT performed the data analysis. JinjZ, YG, and JL drafted the manuscript. All other authors contributed to data collection and collation and approved the final version before submission.

### Conflict of Interest

The authors declare that the research was conducted in the absence of any commercial or financial relationships that could be construed as a potential conflict of interest.
